# A Rare Cause of Abdominal Pain: Scar Endometriosis

**DOI:** 10.1155/2019/2584652

**Published:** 2019-04-17

**Authors:** Banu Karapolat, Hatice Kucuk

**Affiliations:** ^1^Department of General Surgery, Kanuni Training and Research Hospital, Trabzon, Turkey; ^2^Department of Pathology, Kanuni Training and Research Hospital, Trabzon, Turkey

## Abstract

**Introduction:**

Scar endometriosis (SE) is a rare pathology that develops in the scar tissue formed on the anterior abdominal wall usually after a cesarean section. There have been instances of women presenting to emergency or general surgery clinics with abdominal pain due to SE.

**Materials and Methods:**

This study retrospectively reviews 19 patients who were operated on in our clinic between January 2010 and January 2017 with a prediagnosis of SE and were reported to have SE based on their pathology results.

**Results:**

The mean age of the patients was 30.8 years (range: 20-49 years). The body mass indexes of 12 (63.2%) patients were ≥ 25. All patients had a history of cesarean section and 9 (47.4%) patients had undergone cesarean section once. With the exception of one patient who had her SE localized in her inguinal region, all patients had a mass localized on their anterior abdominal wall neighboring the incision and complained about cyclic pain starting in their premenstrual periods. The complaints began 2 years after their cesarean section in 10 (52.6%) patients. Mostly abdominal ultrasonography was used for diagnostic purposes. The lesions were totally excised and the SE diagnosis was made through a histopathological examination in all patients. No postoperative complications or recurrences were seen in any of the patients.

**Conclusion:**

Suspicion of SE is essential in women of reproductive age who have a history of cesarean section and complaints of an anterior abdominal wall mass and a pain at the scar site that is associated with their menstrual cycle. An accurate and early diagnosis can be established in such patients through a careful history and a good physical examination and possible morbidities can be prevented with an appropriate surgical intervention.

## 1. Introduction

Scar endometriosis (SE) is a relatively uncommon entity that usually develops in the skin, subcutaneous tissues, and abdominal and pelvic wall musculature at the site of a surgical scar that occurs after various obstetric or gynecologic surgeries and particularly after a cesarean section [1, 2]. Among the theories put forward to explain the etiology of SE, the most widely accepted one is the iatrogenic direct implantation theory, which asserts that the endometrial cells that detach from the uterus during the surgery become inoculated at the edge of or inside the operation scar [3, 4]. The common symptoms are a mass in the abdominal wall and a cyclic pain associated with menses. When palpated, this mass can be confused with lipoma, abscess, hematoma, hernia, granuloma, desmoid tumor, or sarcoma [5, 6]. For this reason, the anamneses of patients should be questioned well, their history of a cesarean section should be revealed, and care should be taken to find out if their pain is of cyclic nature. Although abdominal ultrasonography (USG), computed tomography (CT), and magnetic resonance imaging (MRI) produce nonspecific information, they are helpful in making a diagnosis [7]. The curative treatment is the excision of the mass, which also allows making a definitive diagnosis of SE through histopathological examination. This study retrospectively reviewed patients who were monitored and treated for SE diagnosis in our clinic and the results obtained were presented by also referring to the literature.

## 2. Materials and Methods

### 2.1. Patients and Study Protocol

This study reviewed 19 consecutive Caucasian patients, who were operated on with a prediagnosis of SE in the General Surgery Clinic in Trabzon Kanuni Training and Research Hospital, Turkey, between January 2010 and January 2017, and whose pathology results confirmed SE. The demographic characteristics, anamneses, the number of cesarean sections undergone, complaints of the patients, the onset of these complaints, the localization and size of the mass, the diagnostic methods used, surgical treatment procedures used, the duration of hospital stays, and patient outcomes were all recorded.

The protocol of this study was approved by the local ethics committee and all patients signed a written consent form. The study was conducted in accordance with the principles in the Helsinki Declaration as revised in 2000.

### 2.2. Statistical Analysis

All statistical data analyses were performed using the Statistical Package for Social Sciences (SPSS), version 15.0, for Windows (SPSS Inc., Chicago, IL, USA). Descriptive statistics were used for comparisons.

## 3. Results

The mean age of the 19 female patients was 30.8 years (range 20-49 years). The body mass indexes (BMI) of 12 (63.2%) patients were ≥ 25, and those of 7 (36.8%) < 25 (median: 26 (IQR: 23-29)). All patients underwent cesarean sections, 9 (47.4%) patients once, 6 (31.6%) patients twice, and 4 (21.0%) patients three times (median: 2 (IQR: 1-2)).

With the exception of one patient who had her SE localized in her inguinal region, all patients had a mass localized on their anterior abdominal wall neighboring the incision and they all complained about cyclic pains starting in their premenstrual periods. SE was embedded in subcutaneous tissues in 17 (89.5%) patients and in muscle layers of abdominal wall in 2 (10.5%) patients. A typical mass felt moderately hard, solid, and partially mobile during palpation and was approximately 2 × 3 cm in size, growing larger during menstruation. The complaints began 1, 2, 3, and 4 years after the cesarean section in 4 (21.1%) patients, 10 (52.6%) patients, 4 (21.1%) patients, and 1 (5.3%) patient, respectively (median: 2 (IQR: 2-3)). SE was found on the right side of the scar in 9 (47.4%) patients, on the left side of the scar in 7 (36.8%) patients, on the midline scar in 2 (10.5%) patients, and in the inguinal region in 1 (5.3%) patient. The SE localized in the inguinal region was close to the medial half of the right inguinal region and also caused cyclic pain.

**Figure 1:**
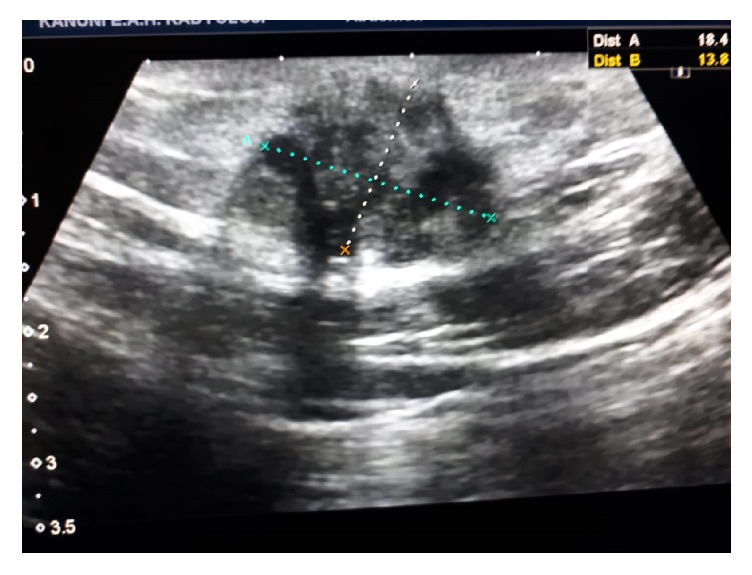
Abdominal USG shows an approximately 18 × 13 mm heterogeneous hypoechoic lesion with lobulated margins, which is localized between subcutaneous tissues and does not indicate vascularization in Doppler examination.

**Figure 2:**
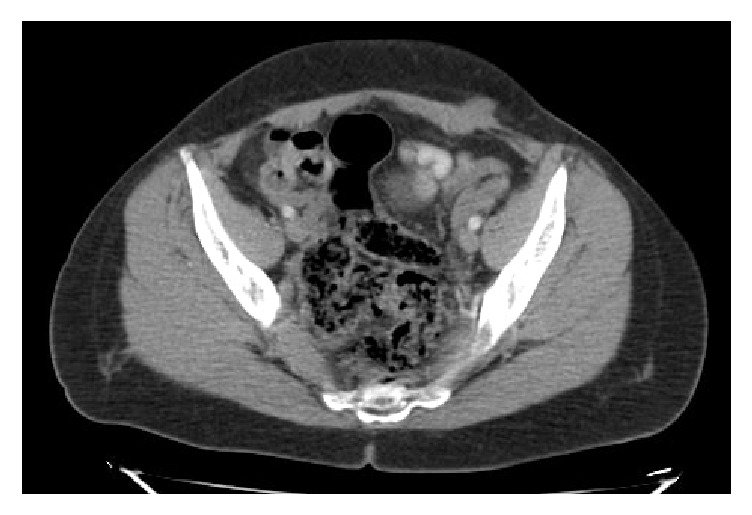
A mass extending in the left rectus abdominis muscle in the lower left abdominal region.

**Figure 3:**
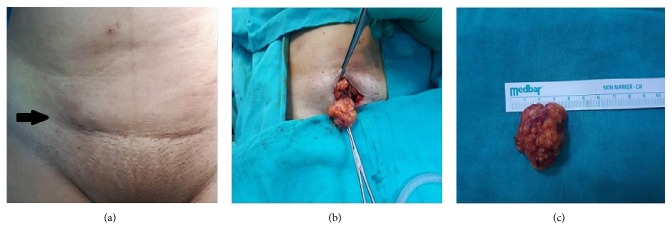
(a) Mass localized on the upper right side of pfannenstiel incision scar (black arrow). (b) A perioperative view of the SE mass. (c) Macroscopic image of the resected mass.

**Figure 4:**
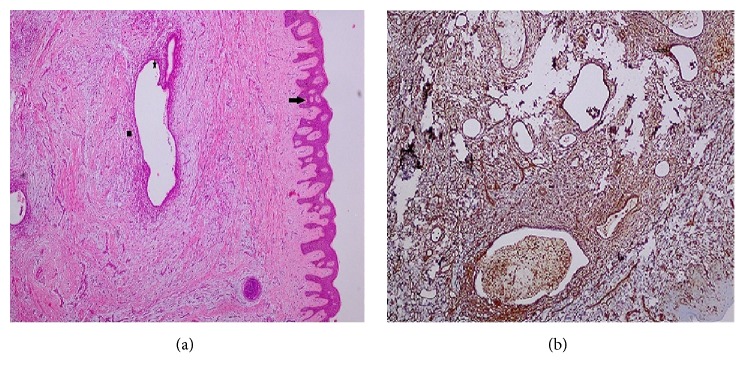
(a) An image of the endometriosis locus in the resected mass. Stratified squamous epithelium (black arrow), endometrial gland (black star), and endometrial stroma (black square) are seen (Hematoxylin-Eosin, original magnification x 4). (b) The endometrial tissue is shown with the arrow in the upper right corner (immunohistochemical staining for Vimentin, original magnification x 10).

All patients had abdominal USG for diagnostic purposes (Figure 1). Additionally, CT was used in 5 (26.3%) patients and MRI in 3 (15.8%) patients (Figure 2). The lesions were totally excised surgically together with at least 1 cm of healthy tissue surrounding them (Figures 3(a), 3(b), and 3(c)). The diagnosis of SE was made through a histopathological examination in all patients (Figures 4(a) and 4(b)). The measurements during the pathological examination showed that the median diameter of the SE masses was 3 cm (IQR: 2.5-3.5). The median duration of hospitalization was 2 days (IQR: 1-3). No postoperative complications were seen in any of the patients. All patients were followed up and no recurrences were encountered in any of the patients (median: 2 years (IQR: 2-4)) All of these abovementioned demographic and clinical characteristics of the patients are summarized in Table 1.

## 4. Discussion

This study underlines five points: (a) SE occurred mostly in women aged around 30 years who had a history of cesarean section, (b) the majority of the patients were obese with a BMI greater than 25, (c) the SE-related complaints began 2 years after the cesarean section in more than half of the patients, (d) the most commonly used diagnostic methods were abdominal USG and CT, and (e) the curative treatment was achieved through surgical mass excision in all patients and no recurrences were seen.

SE is a frequently misdiagnosed pathological condition with an incidence ranging from 0.03 to 1.7% [6]. As general information, SE can be encountered often in women at their reproductive age who have undergone a cesarean section. The mean age of the patients in this study was approximately 30 and all patients had a history of cesarean delivery, mostly once as found in 9 (47.4%) patients. These results are compatible with the information in the literature [8].

The important point in the occurrence of SE is the diligence of the surgeon when performing the surgical procedure. It becomes easier during a cesarean section for amniotic fluid to carry endometrial cells to the skin and subcutaneous tissues. Many obstetric surgeons clean the uterine cavity with dry or wet swabs after a cesarean section. The contact of these swabs with the incision site increases the risk of inoculation and their quick removal from the operation area is necessary to prevent SE occurrence. There are two important points that care should be taken of during the surgery. The first one is forming a physical barrier by placing abdominal compresses on the subcutaneous tissue and the skin before opening the uterine cavity to protect the surgical margins and avoiding the reuse of already used surgical tools such as needle holders and forceps and suture materials when suturing the uterus for the closure of muscles, fascia, subcutaneous tissues, and the skin. The second important point is irrigating the skin, subcutaneous tissues, muscle, and fascia after suturing the uterine cavity by flushing them with pressurized physiological saline solution before continuing with the abdomen closure making certain that no dead space is left in the subcutaneous area. Although the present study did not reveal, due to its retrospective nature, whether the abovementioned protective measures had been taken, we believe that these above speculative practices can prevent endometrial epithelial and glandular cells from being implanted in muscles, subcutaneous tissues, and the skin and hence hinder SE formation.

The fact that majority of the patients in our study had BMIs of 25 and greater suggests that SE incidence may be higher in obese women. Since the subcutaneous fatty tissue on the anterior abdominal wall is thicker and covers a larger area in obese patients, this may constitute a facilitating factor for the implantation of endometrial tissues.

In this study, the complaints of SE patients started mostly 2 years after their cesarean section. This may give an idea on the time it takes for the endometrial cells, glands, and stroma that are implanted during a cesarean section to localize in skin and subcutaneous tissues, proliferate, form a mass, and, after reaching a certain size, respond to the ovarian hormone stimulation during a menstrual cycle, resulting in swelling and cyclic pain.

In the single case where SE was localized in the inguinal region, the distance between the incision and the site of localization suggests that the SE formation in this patient did not occur through implantation but a hematogenous or lymphatic dissemination [9].

In patients whose SE diagnosis is doubtful, other pathologies including lipoma, incisional hernia, suture granuloma, and abdominal wall tumors should be considered for differential diagnosis [6, 10, 11]. In such a case, additional radiological procedures should be employed for the diagnosis. The first choice is abdominal USG, a fairly practical and easily accessible method, which provides information on the size, location, margins, and internal structure of the lesion [11–13]. In USG scans, SE lesions usually appear as heterogeneous, hypoechoic, solid, and irregularly marginated round/oval nodules. Besides helping the diagnosis, CT and MRI may reveal the association of the mass with the abdominal cavity and play an important role in the exclusion of other lesions during differential diagnosis [13]. Mostly USG followed by CT or MRI was used at the stage of diagnosis also in our study. Using USG alone without a subsequent CT or MRI would not produce a definitive diagnosis and would involve the risk of missing out other pathologies. CT and MRI were very helpful in revealing the localization and size of the mass palpated at the anterior abdominal wall, its relationship with the surrounding tissues, and whether there were any other pathologies in the abdomen [6, 13]. We believe that CT or MRI scans should be used more actively in patients who have had USG but whose SE diagnosis remains suspicious. However, it is not possible to make a final diagnosis using these radiological examinations alone. The definitive diagnosis of SE is made after a histopathological examination of the surgically removed tissue has clearly shown the presence of endometrial smooth muscle cells, stroma, glands, and hemosiderin-laden macrophages in the tissue.

The ultimate treatment is achieved through a total surgical removal of the SE mass together with at least 1 cm of surrounding healthy tissue, without impairing the integrity of the mass. This excision prevents occurrence of potential malignant degeneration or recurrence. The postoperative recurrence is reported to be 1.5-9.1% in the literature; no recurrences were seen in our patients during their follow-up [8]. Owing to the surgical excision performed in all patients in our study, curative treatment was achieved and the definitive diagnosis of SE was made in a histopathological way.

The limitations of this study include its retrospective nature, the small sample size from only one center, and the lack of information as to how long it took to resume regular menstrual cycles after the cesarean section. Further prospective studies would be valuable in contributing to these findings.

## 5. Conclusion

SE should always be considered in women of reproductive age who present with a history of cesarean section, a pain at the scar site that is associated with menstrual cycle, and a mass at the anterior abdominal wall. Accurate and early diagnosis can be achieved in such patients with a careful history and a good physical examination and their quality of life can be improved with a prompt surgical intervention. As the rates of cesarean section constantly increase in recent years, it is possible to encounter SE more frequently in the near future. Therefore, it is important for the prevention of SE to increase and expand education that would raise awareness among obstetricians and gynecological surgeons.

## Figures and Tables

**Table 1:** Detailed information on patients with SE.

Patients (n)	19
Age	30.8 years (range 20-49)
BMI	12 (63.2%) 25 and over
	7 (36.8%) less than 25
Number of cesarean section	9 (47.4%) patients 1
	6 (31.6%) patients 2
	4 (21.0%) patients 3
Complaints	
Mass	19 (100%)
Cyclic pain	19 (100%)
Onset of complaints	
1 year after the cesarean section	4 (21.1%)
2 years after the cesarean section	10 (52.6%)
3 years after the cesarean section	4 (21.1%)
4 years after the cesarean section	1 (5.3%)
SE site	
Right side of the scar	9 (47.4%)
Left side of the scar	7 (36.8%)
Middle line of the scar	2 (10.5%)
Inguinal region	1 (5.3%)
Diagnostic tools	
USG	19 (100%)
CT	5 (26.3%)
MRI	3 (15.8%)
Treatment	
Surgical resection	19 (100%)
Diameter of the mass	Median: 3 cm (IQR: 2.5-3.5)
Duration of hospitalization	Median: 2 days (IQR: 1-3)
Duration of follow-up	Median: 2 years (IQR: 2-4)

(BMI: Body mass index, SE: Scar endometriosis, USG: Ultrasonography, CT: Computed tomography, MRI: Magnetic resonance imaging).

## Data Availability

The data used to support the findings of this study are currently under embargo while the research findings are commercialized. Requests for data, [6/12 months] after publication of this article, will be considered by the corresponding author.
